# Phytogenic Generation of NiO Nanoparticles Using Stevia Leaf Extract and Evaluation of Their In-Vitro Antioxidant and Antimicrobial Properties

**DOI:** 10.3390/biom10010089

**Published:** 2020-01-06

**Authors:** Saiganesh Srihasam, Krishnan Thyagarajan, Mallikarjuna Korivi, Veeranjaneya Reddy Lebaka, Siva Pratap Reddy Mallem

**Affiliations:** 1 Department of Physics, Jawaharlal Nehru Technological University, Anantapur, Anantapuramu 515 002, India; srihasamsaiganesh@gmail.com; 2 Department of Physics, Jawaharlal Nehru Technological University, Pulivendula 516 390, India; ktrjntu@gmail.com; 3 Exercise and Metabolism Research Center, College of Physical Education and Health Sciences, Zhejiang Normal University, Jinhua 321004, China; 4 Department of Food Science and Technology, Yeungnam University, Gyeongsan, Gyeongbuk 38541, Korea; 5 Department of Microbiology, Yogi Vemana University, Kadapa, Andhra Pradesh 516003, India; 6 School of Electronics Engineering, Kyungpook National University, Daegu 41566, Korea

**Keywords:** stevia broth, biogenic preparation, NiO nanoparticles, antibacterial, anti-oxidant activity

## Abstract

In the present study, economically viable NiO nanoparticles were produced by biogenic preparation using stevia leaf broth and their in-vitro antioxidant and antimicrobial activities were evaluated. The properties of the prepared NiO nanoparticles were confirmed by analytical techniques such as Ultraviolet-Visible (UV-Vis), X-ray diffraction (XRD), FE-SEM, and Fourier transform infrared spectroscopy (FTIR) analyses. Morphological studies using scanning electron microscopy (SEM) and transmission electron microscopy (TEM) showed that the size of synthesized nanoparticles ranged from 20 to 50 nm, most of which were spherical and few of which were agglomerated. The role of the biological moieties, which reduce and cap the nanoparticles, was studied using FTIR analysis. The prepared nanoparticles strongly inhibited gram-negative bacteria, which is a camper with gram-positive bacteria and fungi. Furthermore, it performs an effective in-vitro activity through α,α-diphenyl-β-picrylhydrazyl (DPPH) reduction. Thus, it can be concluded that the effective and easy green synthesis process used for NiO nanoparticles provides potential antimicrobial agents against multidrug-resistant microbes.

## 1. Introduction

Recently, green nanoscience and technology have developed to a great extent due to their wide applications in catalysis, sensors, drug delivery, and biomedical purposes. There has been increasing research on the utilization of phytomolecules in the area of green synthesis [1,2]. Thus, several investigators have used various phytochemicals from different biological sources such as roots, fruits, barks, flowers, and leaves. Among them, leaves are a rich source of polyphenols, amides, and proteins, which are utilized in the synthesis of noble metal (Au, Ag, Pd, Pt, and Cu) and metal oxide (TiO_2_, NiO, ZnO, and CdO) nanoparticles (NPs) [3,4]. Among the metal oxide nanoparticles, NiO is a cheap, nontoxic to humans in short-term exposure, abundantly available, and photo-stable material. Moreover, NiO is a P-type semiconductor with a wide bandgap of 3.6 to 4.0 eV and has potential applications in gas sensors, catalysis, battery, supercapacitors, and fuel cell applications [4]. However, long-term inhalation of NiO is damaging to the lungs, causing lesions and, in some cases, cancer. The calculated half-life of dissolution of NiO in blood is more than 90 days [5,6,7]. NiO-NPs have been synthesized by using different methods, including chemical precipitation, electrodeposition, hydrothermal, solvothermal, thermal decomposition, microemulsion technique, coprecipitation methods, and microwave irradiation [8,9,10,11,12,13,14]. Nevertheless, these processes have disadvantages owing to the involvement of high pressure, high temperature, and hazardous chemicals. The biosynthetic process is gaining interest since it avoids high costs, several synthesis steps, usage of toxic chemicals, and harsh atmospheric conditions for reduction and stabilization. NiO nanoparticles are prepared using different plant leaf extracts such as *Azadiricta indica*, *Calotropis gigantea, Agathosma betulina*, and *Aegle marmelos* [4,15,16,17]. The leaf extracts from natural sources of polysaccharides, flavonoids, terpenoids, polyphenols, glycosides, proteins, and vitamins act as reducing and capping agents in the preparation of nanoparticles [18].

*Stevia rebaudiana* Bertoni is a sweet herb belonging to the composite family and is native to South America. Its leaf extract is used as an alternative to table sugar and as a supplement in soft drinks, soy sauce, soju, yogurt, and other foods, in Korea, Brazil, and Japan. Stevia extract contains sugars, amino acids, lipids, chlorophylls, alkaloids, flavonoids, xanthophylls, hydroxycinnamic acids, and trace elements [19]. The leaf extract of stevia has been thought to provide health benefits such as anti-hypertensive, anti-hyperglycemic, and antihuman rotavirus activities [20,21]. It has been used extensively for the synthesis of silver, gold, and palladium NPs [22,23,24]. Terpenoids, polyphenols, proteins, and aldoses have been proposed to play a significant role in the formation (reducing and capping) of metal NPs.

Herein, the NiO-NPs were synthesized by using stevia extract as a reducing and capping agent and the structural features of the prepared materials were studied using the X-ray diffraction (XRD) analysis. The morphological analysis was performed using scanning electron microscopy (SEM) and transmission electron microscopy (TEM). The chemical composition and oxidation state of the elements were analyzed through the X-ray photoelectron spectroscopy (XPS) studies. In vitro antioxidant studies and evaluation of the synthesized NiO nanoparticles showed that they could be used primarily in medicinal applications as antibacterials against gram-positive and gram-negative bacteria.

## 2. Experimental Details

### 2.1. Materials and Methods

#### Preparation of Nickel Oxide Nanoparticles Using Stevia Leaf Broth

The analytical grade reagent of nickel acetate was procured from Sigma-Aldrich (MO, USA) and used to avoid further purification. The stevia leaves were collected and washed thoroughly with distilled water and dried under shade. The stevia broth was prepared using 5 g of crushed leaves and the addition of 100 mL distilled water in a 200 mL conical flask, which was then boiled for 2 min before decanting it. The boiled broth was filtered with a Whatman no 1 (Pore size: 20 μm) filter paper and used for phytogenic generation of NiO nanostructures. The NiO nanostructures were produced by treating 1 g of nickel acetate in 200 mL distilled water with 25 mL aqueous stevia extract and stirring for 2 h to attain a homogeneous solution. The prepared mixture was then heated at 100 °C until the water evaporated, and the resulting sample was annealed at 500 °C for 2 h. The schematic representation of NiO NPs preparation is described in Figure S1 (Supplementary Materials).

### 2.2. Analytical Techniques for Analysis

The optical properties of the prepared nanostructures were studied using the Ultraviolet-Visible (UV-Vis) spectrophotometer (Thermo Scientific Genesys 10S, Marietta, OH, USA); the phase formation of the prepared materials was analyzed through XRD (PANalytical, Xpert^3^, Malvern, UK); the morphological features such as size and shape of the nanoparticles were examined through SEM (Hitachi S-4800, Hitachi High Technologies Corporation, Tokyo, Japan) and TEM (Technai G2 F 20 S Twin, Hillsboro, OR, USA); and the functional groups of the extract, which play an active role in the synthesis of nanoparticles, were examined through Fourier transform infrared spectroscopy (FTIR, Nicolet iS50, Thermo Scientific, Marietta, OH, USA) [24]. The chemical composition and elemental oxidation states were analyzed through the XPS (Thermo Scientific Al-Kα, Marietta, OH, USA).

### 2.3. Antioxidant Activity Studies

The antioxidant activity (AA%) of NiO-NPs was determined by DPPH free radical scavenging capacity as described by Brand-Williams et al. [25]. The reaction mixture was comprised of 0.5 mL of NiO-NPs (10–200 ug/mL), 3 mL of absolute ethanol, and 0.3 mL of DPPH solution (prepared by dissolving 0.5 mM in ethanol). The reaction between DPPH and the hydrogen donating antioxidant compound resulted in the reduction of DPPH, and led to the color change from deep violet to light yellow. The absorbance (Abs) of the resulting product was read at 517 nm after 30 min of dark incubation. The blank contains ethanol (3.3 mL) and a sample (0.5 mL). The control comprises ethanol (3.5 mL) and DPPH radical solution (0.3 mL). The scavenging activity percentage (AA%) was calculated using the following equation:

Scavenging activity = ((A_blan517_ − A_sample517_)/A_blank517_) × 100 (1)

### 2.4. Antibacterial Activity Studies

Antimicrobial activity of the prepared NiO-NPs was determined using Gram-negative (*E. coli*) and Gram-positive (*Bacillus sublitis* and *Streptococcus pneumonia*) bacteria and fungi *Aspergillus niger* and *Aspergillus fumigatus*. The microbial cultures were collected from Microbial Type Culture Collection Center (MTCC, Chandigarh, India). The disc diffusion method was used to study the microbial inactivation potential of NiO-NPs. Two hundred microlitres of overnight bacterial cultures were spread on Petri platesprepared with 20 mL of sterile LB agar (LBA, Himedia, India). The fungal spores were spread on Potato dextrose agar (PDA, Himedia, India). Ten microlitres of 0.1 mg/mL NiO-NPs were dissolved in DMSO and utilized to test the microbial inactivation potential. The inhibition zone diameter (mm) was measured using Vernier calipers after incubating the bacteria for 12 h and the fungi for 72 h at 37 °C.

## 3. Results and Discussion

### 3.1. UV-Vis Absorption Studies

**Figure 1:**
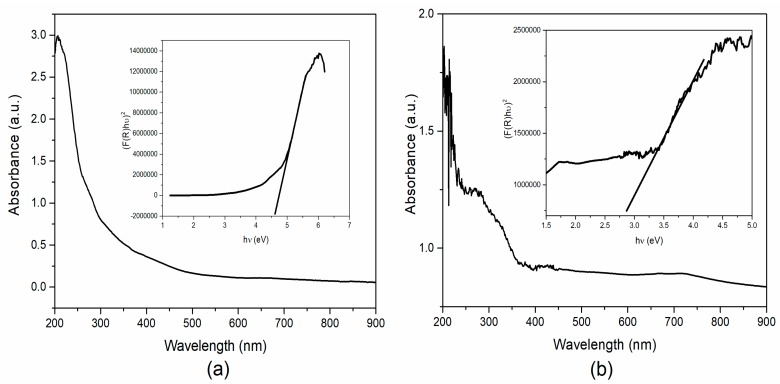
Ultraviolet Visible (UV-Vis) absorption and bandgap measurement of the (**a**) as-prepared and (**b**) annealed NiO nanoparticles.

For optical properties, the Ultraviolet-Visible-Diffuse reflectance spectra (UV-Vis–DRS) was recorded as depicted in Figure 1a,b. The bandgap of the materials was calculated from these results by using the Kubelka–Munk (KM) equation [23]. From the KM equation and the plot between the photon energy and [F(R)hν]^2^, the bandgap was calculated using the Tauc plot (inset of Figure 1a,b). The bandgap of the as-prepared NiO nanostructures was calculated to be around 4.6 eV, and after annealing the sample, the bandgap was shown in the range of 2.7 eV. The higher bandgap (lower absorption band < 300 nm) of NiO was due to the electron transition from ionized oxygen deficiencies to the valance band of NiO arising from the surface defects. In the case of annealed NiO, the bandgap was altered due to the absorption of oxygen, which reduced oxygen defects in the sample. The band gap of NiO nanoparticles changed from around 4.6 to 2.7 eV after annealing the sample, which may have been due to the formation of NiO and desorption of H_2_O from the Ni(OH)_2_ structures. The fabricated bandgap of NiO nanoparticles was in good agreement with recently reported literature [15,26].

### 3.2. XRD Analysis

**Figure 2:**
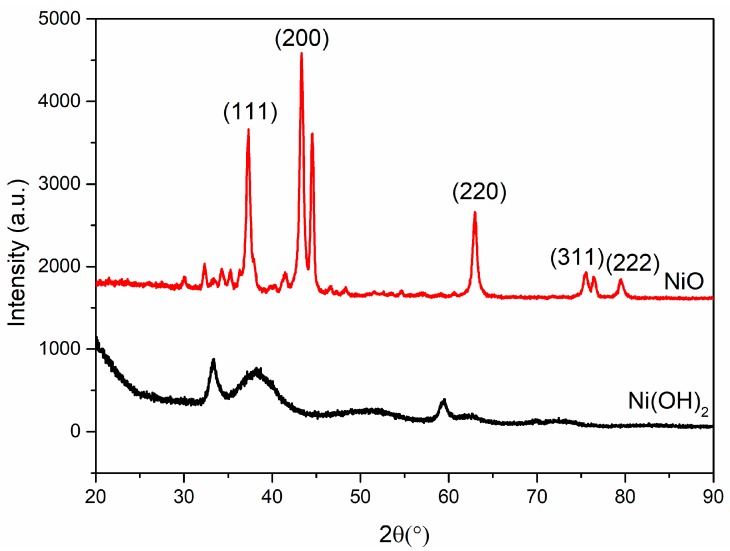
X-ray Diffraction (XRD) spectra of NiO nanostructures.

In order to investigate the phase purity of the prepared materials and the annealed (500 °C) sample, XRD analysis was performed and the results are presented in Figure 2. In XRD diaphragm, the Braggs diffraction peaks were observed at 37.31, 43.35, 62.99, 75.58, and 79.52, which are phase indexed with the face-centered cubic (111), (200), (220), (311), and (222) facets, respectively. The obtained well-matched with JCPDS no: 1313-99-1 with the Fm3m space group has a lattice parameter of *a* = 4.1771 Å [24]. Moreover, there were some additional peaks in the XRD, which may be impurity peaks owing to the biomolecules. Furthermore, the average crystallite size of the NiO-NP was calculated by using the following Scherrer’s equation:

*D* = *Kλ*/*βcosθ*

where *D* is the crystallite size (nm), *K* is the shape factor 0.9, *λ* is the wavelength of the incident (nm), *β* is full width at half maximum, and *θ* is peak position of the corresponding facet. The evaluated average crystallite size of the nanoparticle was 13.7 nm using high-intensity Braggs peak of (200).

### 3.3. SEM and TEM Analysis

**Figure 3:**
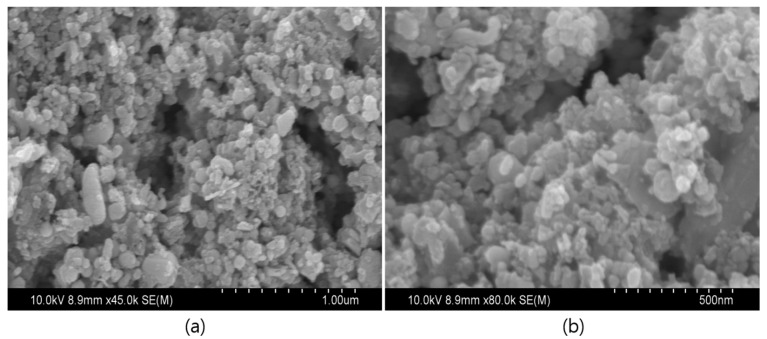
Scanning Electron Microscopy (SEM) images of NiO nanostructures (**a**) as-prepared and (**b**) annealed.

**Figure 4:**
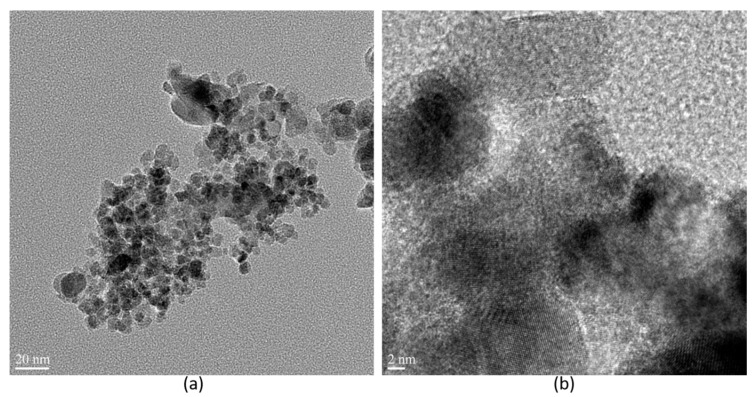
Different Magnification Transmission Electron Microscopy (TEM) images of NiO nanostructures (**a**) 20 nm (**b**) 2 nm.

The morphological features of the prepared NiO nanostructures were studied using SEM, as represented in Figure 3. The results showed that the prepared NiO nanostructures were spherical with sizes ranging between 10 and 40 nm. Moreover, a few of them were slightly agglomerated, which may be due to the large volume of heat produced in the annealing process. However, the small-sized particles were very reactive due to sharp edges, which contain a high surface-to-volume ratio (the higher portion of outward atoms) and less cohesive energy compared with bulk materials [27,28]. Furthermore, the particle size, shape, and distribution of the synthesized nanoparticles were determined using HR-TEM analysis and their lattice fringes were evaluated. The resulting TEM images and their lattice fringes are presented in Figure 4a,b, respectively. Figure 4a depicts the size of the NiO nanoparticles in the range of 7–40 nm with a spherical shape. Most of the particles were very small, and well distributed, and very few particles were agglomerated. From Figure 4b represents the highly crystalline nature with an interplanar spacing of 0.239 nm for (111) plane of NiO nanostructures. In addition to that, the particle size, shape, and crystalline structure of nanoparticles influence the antimicrobial activity [29,30,31,32]. Muhammed et al. [30] have been noticed that the smallest-sized spherical AgNPs demonstrated better antibacterial activity against bacterial strains, compared to the triangular and larger spherical-shaped AgNPs. One of the reasons for the higher antibacterial activity of these anisotropic-shaped AgNPs may be the basal plane with high-atom-density (111) facets, which act as the maximum reactivity sites leading to the strongest antibacterial activity. Large surface-to-volume ratio in spherical shapes may be the reason why maximum reactivity obtains the highest antibacterial activity. In addition to this, Anon [31] compared the antibacterial activity of Ag, CuO, NiO, and ZnO nanoparticles and confirmed that the spherical and small size Ag-NPs had higher antibacterial activity. With floral CuO and rod-like CuO nanoparticles, higher antibacterial activity was observed in floral CuO. Compared to the different shapes and sizes, the octahedral NiO showed the highest antimicrobial activity. This may be because, due to their shape and crystal quality, fine-crystalline-structured NiO nanoparticles are more reactive. Even though the petals of the floral copper oxide, rod-like copper oxide, and octahedral nickel oxide nanoparticles are bigger in size (in m) than the silver nanoparticles (in nm), the shape is takes charge of the microbial activity and shows excellent antimicrobial activity equal to that of Ag nanoparticles. From the above information, one can conclude that shape plays a more vital role than the size of the nanoparticles. Further investigations have to be carried out for the synthesis of small-sized different shaped nanoparticles that could provide a far better understanding of the mechanism of metal oxide nanoparticle’s antimicrobial activity.

### 3.4. FTIR Analysis

**Figure 5:**
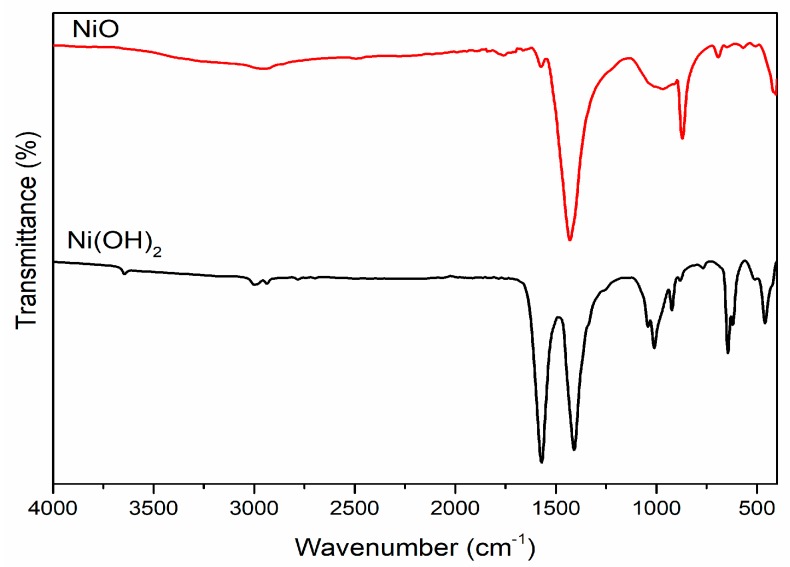
Fourier transfrom infrared spectroscopy (FTIR) spectra of NiO nanostructures.

To examine the functional groups, which act as reducing and capping agents of the prepared NiO nanostructures, FTIR spectroscopy was performed, and the results are presented in Figure 5. The prepared mixture was then heated at 100 °C until the water evaporated, and the resulting sample was annealed at 500 °C for 2 h in ambient atmosphere. In the case of as-prepared Ni(OH)_2_ nanoparticles, the vibrational peaks were observed at 3640, 3005, 2934, 1566, 1407, 1000, 908, 645, and 458 cm^−1^. The peak at 3640 cm^−1^ corresponded to H-bonded phenols and 3005 and 2934 cm^−1^, suggesting OH and H-bonded carboxylic groups [33]. The doublet peaks 1566 and 1407 cm^−1^ were distinctive N-H stretching vibrations of the amide linkage of the proteins. Moreover, the peaks between 1000 and 400 cm^−1^ represented structural vibrations of Ni-OH and Ni-O-Ni. Moreover, in the case of NiO (annealed at 500 °C) nanostructures, the peaks were observed at 2953, 1574, 1426, 964, 873, 684, and 406 cm^−1^ [34]. The slight shift in the characteristic vibration at 1574 cm^−1^ may be due to the attachment with NiO and intensity of 1426 cm^−1^ increase owing to the amide group of protein linkage with the surface of the NiO surface. The peaks at 964 and 684 cm^−1^ were distinctive modes of the Bunsenite NiO phase.

### 3.5. XPS Studies

**Figure 6:**
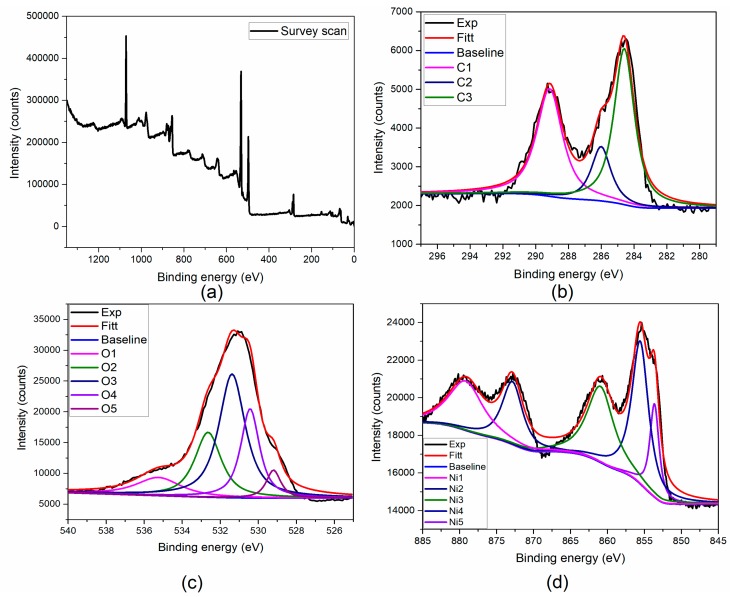
X-ray photoelectron spectroscopy (XPS) analysis of NiO nanostructures (**a**) Survey scan (**b**) Carbon (**c**) Oxygen (**d**) Nickel elements.

The chemical composition and elemental electronic structure of the prepared NiO structures were analyzed using the XPS analysis, and the results are depicted in Figure 6. The survey scan in Figure 6a showed the presence of C, O, Ni, and N elements in the prepared material. The deconvoluted enlarged spectra of C1s (Figure 6b) depict the peaks C1, C2, and C3 at 289.1, 285.6, and 284.5 eV, which were attributed to the O-C=O, C-OH/C-O-Ni, and C-C/C=C. The peaks of 289.1 eV denote the carboxyl molecules, 285.6 eV represents the presence of residual oxygen-containing groups and C-O-Ni linkages, and 284.5 eV represents the non-oxygenated carbon (C-C/C=C) of benzene rings of the biomolecules, which are present in the stevia extract for reducing and capping of NiO nanoparticles during synthesis. The high-resolution spectra of O1s are depicted in Figure 6c; the spectrum has five deconvoluted peaks at 535.0 (O1), 532.6 (O2), 531.4 (O3), 530.4 (O4), and 529.3 eV(O5). The peak position at 529.3 was attributed to the oxygen in NiO, and 530.4 corresponds to the formation of Ni-O-Ni/C-O-Ni through the oxygen-containing functional groups of the biomolecules, which act as capping materials of NiO formation. Moreover, the peak at 531.4 was attributed to the C=O group or shoulder peak of NiO. Furthermore, the peaks of 532.6 and 535.0 eV represent Ni-O-H bonds and H_2_O adsorbed on the surface of the NiO nanoparticles [35]. The broad-spectrum Ni (Figure 6d) represents the characteristic peaks of Ni^2+^ 2p_3/2_, and its satellite peaks were found at 853.4, 855.6, and 861.0 eV, respectively, while the Ni 2+2p1/2 peak was observed at 872.9 and its satellite peak was located at the position of 879.2 eV, which strongly confirmed the formation of NiO structures.

### 3.6. Biological Studies: Antioxidant and Antimicrobial Activities

Phytogenic NiO-NPs showed strong antioxidant activity through the DPPH reduction assay. NiO-NPs showed dose-dependent scavenging capacity, and the highest value of 70% was observed for 200 μg/mL. DPPH reduction capacity of NiO-NPs at different concentrations was shown in Figure S2. DPPH free radical quenching in a time-dependent manner occurring through the interchanging of electrons between the reactants and formed products is responsible for the antioxidant property of the phytogenic NiO-NPs [25,36].

**Figure 7:**
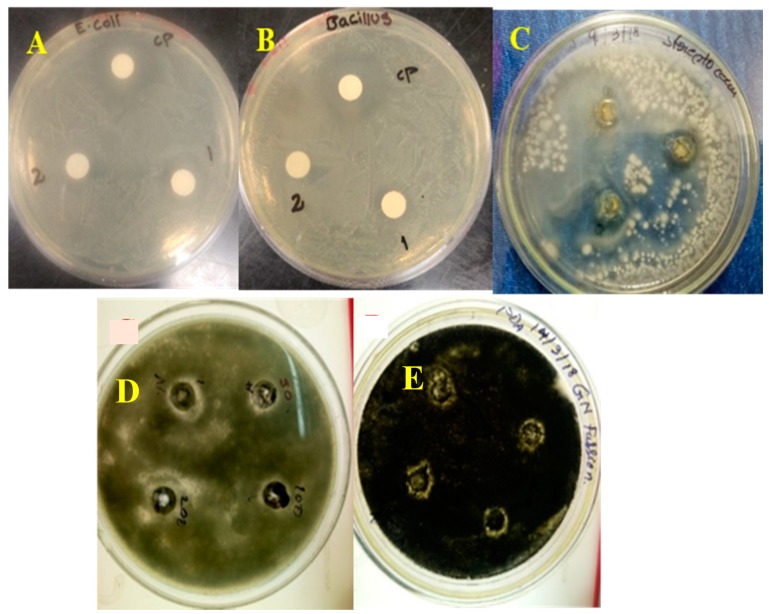
Antimicrobial activity against (**A**) *E. coli*, (**B**) *B. subtilis*, (**C**) *S. pneumonia* (**D**) *A. niger*, and (**E**) *A. fumigatus.*

Antimicrobial activity of the stevia leaf broth NiO-NPs was assessed against three bacterial and two fungal strains, and the measured values of the inhibition zones (mm) are as shown in Table 1 and Figure 7. From the data, the phytogenic NiO-NPs were more effective against Gram-negative bacteria, *Escherichia coli* (16 mm), when compared to Gram-positive bacterial strains *Bacillus sublitis* (15) and *Streptococcus pneumonia* (14 mm), and fungi *A. niger* (6) and *A. funigatus* (4). Generally, the microbial inhibition potential was higher against the Gram-negative bacteria than the Gram-positive bacteria and fungi due to the absence of a peptidoglycan layer in the Gram-negative bacteria, whereas the Gram-positive bacteria had a thick peptidoglycan layer and the fungi were more resistant to environmental conditions and had a strong chitinous membrane layer for protection [18,37]. These results were contrary to previous reports of NiO-NPs synthesized through biogenic/phytogenic preparation [4,15,16,17].

**Figure 8:**
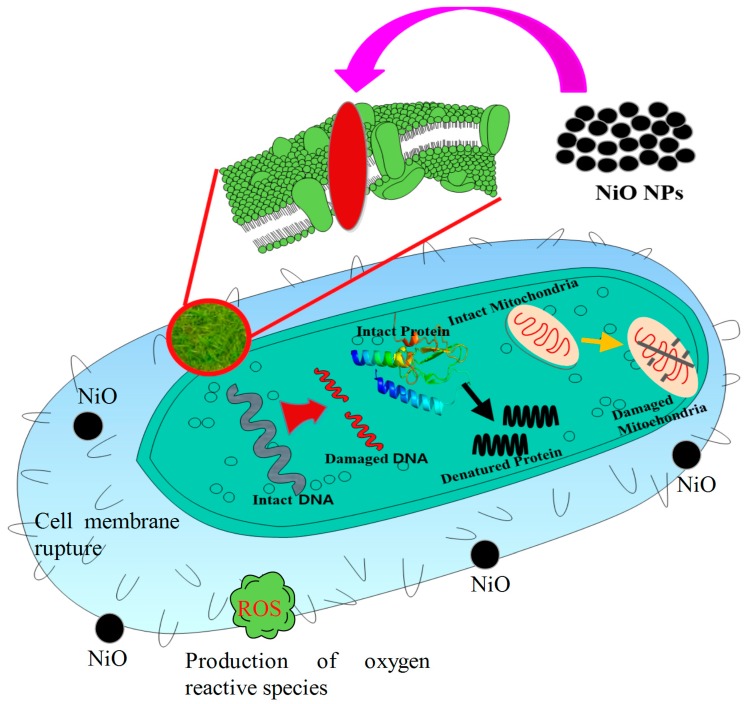
The hypothetical antimicrobial mechanism of NiO nanoparticles.

Due to their minute size and stability, the NiO-NPs showed enhanced antibacterial activity in the present study. Smaller size usually enhanced the dispersibility and penetration into the intracellular matrix and interfered with the intracellular Ca^2+^ absorption and caused cell damage. Attachment of the nanoparticles with decreased size initiated electrostatic interactions between the Nickel ions released from the NiO-NPs and the cell membrane of the microbes, which led to cell membrane damage [38]. The damaged cell membrane is more sensitive to further interactions and allows for the penetration of nanoparticles and leakage of the intracellular organelles. These sequential steps enhance the inhibitory activity of the nanoparticles. The hypothetical antimicrobial mechanism of the NiO-NPs is shown in Figure 8. Penetration of NiO-NPs inside the microbial cell further improves the microbial inhibition by interacting with the electron transport and damaging the DNA by breaking the phosphate and hydrogen bonds, denaturing the protein by altering the tertiary structure, and damaging the mitochondria by oxidative stress ROS generated by the interactions between the inorganic metal and metal oxide nanoparticles, which finally lead to cell death. The potential of activity is mainly governed by the size, shape, dosage, stability, morphology of the nanoparticles, and treatment time [39]. Since the activity and stability of NiO nanoparticles are very effective against the common pathogens, their utility in commercial paintings kills the airborne pathogens with high efficiency and can also be utilized as an effective catalyst in wastewater treatment instead of the bleaching process.

## 4. Conclusions

In conclusion, we have successfully produced economically viable NiO-NPs by using stevia leaf broth and evaluated their in-vitro antioxidant and antimicrobial activities. The size of the prepared NiO-NPs ranged between 7–40 nm with a spherical shape. The stevia-synthesized NiO-NPs were more effective against Gram-negative bacteria, *Escherichia coli* (16 mm) than the Gram-positive bacteria, *Bacillus* (12 mm), *Streptococcus Pneumonia* (14 mm). Their utility in commercial paints kills the airborne pathogens with high efficiency and can also be used as an effective catalyst in wastewater treatment to replace the bleaching process. The antifungal activity of the NiO-NPs could be applied in the field of biopesticides, where these can be applied as an alternative to environmentally toxic chemical pesticides.

## Figures and Tables

**Table 1:** Zone of inhibition (mm) measurements for the five selected microbes.

Compd.	Zone of Inhibition (mm)				
	*E. coli*	*B. subtilis*	*S. pneumonia*	*A. niger*	*A. fumigatus*
NiO	16	12	14	6	4
Ni(OH)_2_	20	10	16	8	6
Chloramphenicol	28	26	20	12	10

## Supplementary Materials

The following are available online at https://www.mdpi.com/2218-273X/10/1/89/s1, Figure S1: Schematic representation of NiO NPs using Stevia leaf extract, Figure S2: DPPH reduction pattern of NiO-NPs at different concentrations.
