# Supplementary material for: Phytogenic Generation of NiO Nanoparticles Using Stevia Leaf Extract and Evaluation of Their In-Vitro Antioxidant and Antimicrobial Properties
Source: Biomolecules. 2020 Jan 6;10(1):89. doi: 10.3390/biom10010089 (PMC7023445; doi:10.3390/biom10010089)
Supplement: Supplementary file 1 [file biomolecules-10-00089-s001.pdf]

Supplementary Material

Phytogenic Generation of NiO Nanoparticles Using Stevia Leaf Extract and Evaluation of Their In-Vitro Antioxidant and Antimicrobial Properties

Saiganesh Srihasam [1,†], Krishnan Thyagarajan [2,†], Mallikarjuna Korivi [3,*], Veeranjaneya Reddy Lebaka [4,5,*] and Siva Pratap Reddy Mallem [6,*]

[1] Department of Physics, Jawaharlal Nehru Technological University, Anantapur, Anantapuramu 515 002, India; srihasamsaiganesh@gmail.com
[2] Department of Physics, Jawaharlal Nehru Technological University, Pulivendula 516 390, India; ktrjntu@gmail.com
[3] Exercise and Metabolism Research Center, College of Physical Education and Health Sciences, Zhejiang Normal University, Jinhua 321004, Zhejiang, China
[4] Department of Food Science and Technology, Yeungnam University, Gyeongsan Gyeongbuk 38541, Korea
[5] Department of Microbiology, Yogi Vemana University, Kadapa, Andhra Pradesh-516003, India
[6] School of Electronics Engineering, Kyungpook National University, Daegu 41566, Korea
* Correspondence: mallik.k5@gmail.com (M.K.); lvereddy@yahoo.com (V.R.L.); dr.mspreddy@gmail.com_ (S.P.R.M.)
† These authors contributed equally to this work.

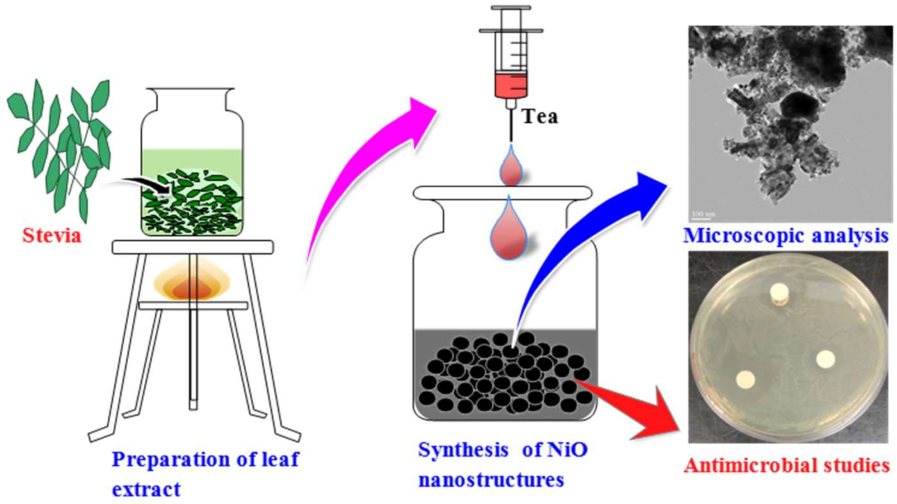

Figure S1. Schematic representation of NiO NPs using Stevia leaf extract.

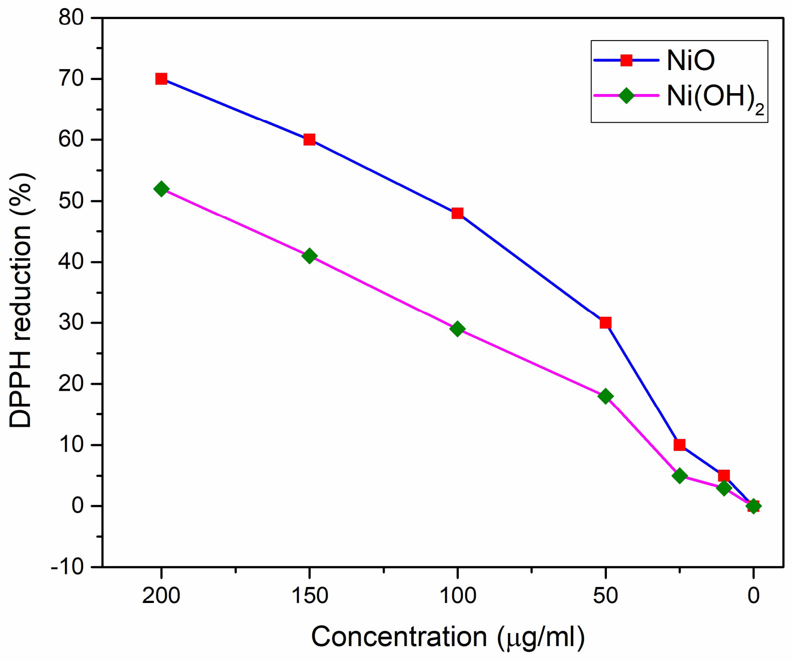

Figure S2. DPPH reduction pattern of NiO-NPs at different concnetrations.
